# Urine output as one of the most important features in differentiating in-hospital death among patients receiving extracorporeal membrane oxygenation: a random forest approach

**DOI:** 10.1186/s40001-023-01294-1

**Published:** 2023-09-15

**Authors:** Sheng-Nan Chang, Nian-Ze Hu, Jo-Hsuan Wu, Hsun-Mao Cheng, James L. Caffrey, Hsi-Yu Yu, Yih-Sharng Chen, Jiun Hsu, Jou-Wei Lin

**Affiliations:** 1 Cardiovascular Center, National Taiwan University Hospital Yunlin Branch, Douliu City, Yunlin County Taiwan; 2 Department of Medicine, College of Medicine, National Taiwan University, Taipei, Taiwan; 3 Department of Information Management, National Formosa University, Huwei, Yunlin Taiwan; 4 Shiley Eye Institute, University of California San Diego, La Jolla, CA USA; 5 Office of Medical Informatics, National Taiwan University Hospital Yunlin Branch, Douliu City, Yunlin County Taiwan; 6 Physiology and Cardiovascular Research Institute, University of North Texas Health Science Center, Fort Worth, TX USA; 7 Department of Surgery, National Taiwan University Hospital, Taipei, Taiwan; 8 Department of Surgery, College of Medicine, National Taiwan University, Taipei, Taiwan; 9 Department of Surgery, National Taiwan University Hospital Yunlin Branch, Douliu City, Yunlin County Taiwan

**Keywords:** Extracorporeal membrane oxygenation, Machine learning algorithm, Random forest, Oliguria

## Abstract

**Background:**

It is common to support cardiovascular function in critically ill patients with extracorporeal membrane oxygenation (ECMO). The purpose of this study was to identify patients receiving ECMO with a considerable risk of dying in hospital using machine learning algorithms.

**Methods:**

A total of 1342 adult patients on ECMO support were randomly assigned to the training and test groups. The discriminatory power (DP) for predicting in-hospital mortality was tested using both random forest (RF) and logistic regression (LR) algorithms.

**Results:**

Urine output on the first day of ECMO implantation was found to be one of the most predictive features that were related to in-hospital death in both RF and LR models. For those with oliguria, the hazard ratio for 1 year mortality was 1.445 (*p* < 0.001, 95% CI 1.265–1.650).

**Conclusions:**

Oliguria within the first 24 h was deemed especially significant in differentiating in-hospital death and 1 year mortality.

**Supplementary Information:**

The online version contains supplementary material available at 10.1186/s40001-023-01294-1.

## Introduction

Patients with severe heart or lung failure can benefit from extracorporeal membrane oxygenation (ECMO). ECMO was required for critically ill patients with guarded prognoses regardless of the initial etiology [1]. Taiwan's national registry reports that the overall morality of adults was near 60% after 1 month and 75% after 1 year [2]. At 1 month and 1 year, overall mortality rates were near 30 and 45%, respectively, for patients under 18 years [3]. As an invasive procedure, ECMO is associated with bleeding, embolisms, and infections [2, 3]. Consequently, finding objective criteria to select suitable critical care candidates for ECMO and to identify those likely to require extraordinary measures is crucial [4].

To predict outcomes in patients receiving ECMO support, risk scores have been developed, such as the Survival After Veno-Arterial ECMO (SAVE) Score and the ECMO-ACCEPTS Score [4–8]. The prognosis of using ECMO and the potential to receive subsequent therapy is highly variable among individuals according to their underlying etiologies and baseline pathophysiological conditions. Consequently, the reported discriminatory power (DP) of these approaches varies based on the inclusion criteria, ECMO setup, statistical algorithms, stratification strategies, and system evaluation.

The highly correlated variables included in the analysis could also be a significant reason for this disequilibrium. Despite the fact that these clinical parameters represent damage to different organs, there is interaction between these organs, and they are always interconnected. Due to this, it is vital to clarify this issue using a different method than the traditional one. Using newly developed machine learning algorithms to perform supervised classification, it sheds light on a distinct method of clarifying this issue [9–12].

Machine learning algorithms employ various methods, such as probabilistic and optimization approaches, to learn from past experience and detect useful patterns in large, unstructured and complex data sets [13]. The random forest algorithm (RF) has shown superior accuracy for disease prediction among multiple supervised machine learning algorithms [13]. To determine the most reliable predictor of clinical outcome for ECMO patients, we have applied this novel algorithm and also compared to the conventionally used logistic regression (LR) model.

## Methods

### Setting and participants

Data from the ECMO registry of a single medical center in Taiwan were retrospectively analyzed. All patients on ECMO were included in the analysis. As potential predictive markers of future outcomes, clinical metrics were collected prior to ECMO and early in resuscitation. This study has been conducted in accordance with the ethical standards in the 1964 Declaration of Helsinki. The collection and review of patient information was approved by the Institutional Review Board of National Taiwan University Hospital (#201002034R, 2010/02, ECMO data analysis study).

### Materials

As part of the analysis, demographic, anthropometric, cardiorespiratory, standard laboratory tests, inotropic therapy, urine output, and ventilator settings were used both before and after ECMO placement (Additional file 1: Table S1). Placement of ECMO was classified as cardiovascular, respiratory, or other. A binary ECMO mode was available: veno-arterial (VA) or veno-venous (VV). A single metric labeled inotropic equivalent (IE) was developed by combining dopamine, dobutamine, epinephrine, norepinephrine, isoproterenol, and milrinone in inotropic therapy [14]. Cardiopulmonary resuscitation, intra-aortic balloon pump support, renal replacement therapy, infection, and the Glasgow Coma Scale (GCS) were recorded [1, 4, 5, 7, 15]. It was possible to calculate different risk scores by combining these variables. A total of 55 parameters were available as predicting variables (features). Serial renal function and blood lactate collected afterward in the intensive care unit were not considered early markers and, therefore, were not used. In-hospital mortality during the index hospitalization when ECMO was instituted was the primary endpoint of this study. We recorded the date, time, and cause of death.

### Management of missing data

Missing data points are inevitable in a data set of this size. To avoid listwise deletion in the following analyses, a mean value was substituted for continuous variables or the most frequent value for categorical variables if the absent variable comprised less than 5% of the total values. As per clinical convention, continuous measures with a higher percentage of missing data (> 5%) were categorized into three (normal, abnormal, and missing) or four classes (high, intermediate, low, and missing). Additional file 1: Table S1 shows details of data management for each variable. For every patient, the target outcome variable (in-hospital death) was available.

### Statistics

The baseline characteristics of the patients are reported as a distinct group. We presented continuous variables as means and standard deviations (SD) and categorical variables as percentages. We estimated the probability of in-hospital death using a random forest (RF) and multi-variable logistic regression (LR) model. All predicting variables were included in the "non-parsimonious model," regardless of their statistical significance or potential collinearity. Based on this model, each individual was assigned a risk score. Further receiver operating characteristic (ROC) curve analysis used this "estimator" as the test variable. To identify variables statistically associated with in-hospital death, a novel LR model was applied (stepwise selection, p < 0.05 for inclusion). We developed a parsimonious model using the newly selected variables partitioned as before with recalculated AUC of ROCs.

Feature importance is the degree to which a feature (or a predicting factor) relates to the target outcome. The Gini importance calculated from the RF structure was used to identify the most relevant features among the predicting variables based on the mean decreased "impurity" [16, 17]. To ensure that the select feature also played an imperative role when different models were applied, LR was used again to calculate feature importance [17].

According to the select feature, survival analyses were performed regarding overall mortality up to 1 year, including Kaplan Meier curves, log-rank tests, and Cox regression models. In this study, the duration of ECMO was defined as beginning at the time of its placement and ending at the date of death (event), 1 year after the placement of ECMO, or termination of the study (censored).

IBM SPSS Statistics for Windows, version 24 (IBM Corp., Armonk, N.Y. USA) was used for statistical analysis. Python 3.10.6 (Python Software Foundation, Beaverton, USA) and IBM SPSS Modeler trial version for Windows (IBM Corp., Armonk, N.Y. USA) were used to execute machine learning algorithms.

### Evaluation of system performance by random forest

Patient groups were randomly divided into training and test sets by the following ratios: 75%:25%, 70%:30%, 60%:40%, and 50%:50%. The model was developed using cases from the training set. On the test set, the same model was applied. The area under the curve (AUC) of the ROC analyses in each set was used to determine the DP of the estimator derived from each model.

## Results

During the study period, 1,342 patients underwent ECMO. The mean age was 53.5 years (SD 15.7), and 71.5% were male. It was found that VA mode (83.4%) was used in most cardiovascular cases (80.6%) and VV mode in most of the remaining cases (Additional file 1: Table S2). Table 1 summarizes continuous variables and associated risk scores with Table 2 including categorical metrics. Overall, 62.3% of cases resulted in in-hospital deaths.

**Table 1:** Demographic data of the 1342 patients receiving ECMO, description of variables with valid data

Variables	Units	Valid n	Mean	Standard deviation
Age	years	1342	53.5	15.7
Body height	cm	1342	165.0	8.5
Body weight	kg	1342	68.9	16.5
Body mass index	kg/m2	1342	25.2	5.0
Glasgow coma scale		1342	10.2	5.3
Inotropic equivalent		1342	28.9	44.2
Preset ventilation rate	per minute	1342	17.3	6.1
FiO2		1342	0.9	0.2
Peak inspiratory pressure	cmH2O	1342	27.8	8.8
Peak end-expiratory pressure	cmH2O	1342	7.4	3.9
Mean airway pressure	cmH2O	1342	14.0	5.1
Body temperature	degree Celsius	1342	36.6	1.2
Respiratory rate	per minute	1342	19.2	7.1
Heart rate	per minute	1342	104.2	36.0
Systolic blood pressure	mmHg	1342	95.7	31.4
Diastolic blood pressure	mmHg	1342	57.5	18.1
24 h urine amount	dL	1342	10.8	13.2
pH		1342	7.3	0.2
PaCO2	mmHg	1342	42.2	22.1
PaO2	mmHg	1342	120.7	100.3
PaO2/FiO2	mmHg	1342	166.8	171.1
Bicarbonate	mEq/L	1342	20.1	6.7
Total bilirubin	mg/dL	1035	2.4	4.1
Blood urine nitrogen	mg/dL	1134	37.4	26.8
Creatinine	mg/dL	1342	2.1	1.8
Sodium	mmole/L	1342	139.3	6.9
Potassium	mmole/L	1342	4.4	1.0
Lactate	mmole/L	1068	8.3	6.4
White blood cell	per uL	1342	13,610.9	7335.9
Hematocrit	%	1342	36.3	7.6
Platelet	10^3^/uL	1342	204.8	865.5
Prothrombin time		981	1.5	1.0
Creatine kinase	U/L	864	1489.0	6561.2
Aspartate aminotransferase	U/L	929	503.8	1615.9
Creatine kinase MB	U/L	835	84.5	149.8
Troponin I	ng/mL	665	19.0	174.8
Charlson		929	4.3	3.1
APACHE		998	19.6	8.2
SOFA		841	12.0	4.4
LODS		820	9.9	3.7
MODS		774	9.1	3.7
SAPS3		840	53.2	12.0
SAVE		429	-5.4	5.7

Charlson: Charlson Comorbidity Index, APACHE: Acute Physiologic Assessment and Chronic Health Evaluation II Scoring System, SOFA: Sequential Organ Failure Assessment Score, LODS: Logistic Organ Dysfunction System, MODS: Multiple Organ Dysfunction, SAPS3: Simplified Acute Physiology Score III, SAVE: The Survival After Veno-arterial ECMO Score

**Table 2:** Demographic data of the 1342 patients receiving ECMO, description of categorical data using missing values as a distinct level

Variables	Categories	Valid *n*	%
Sex	Male	959	71.5
	Female	383	28.5
ECMO categories	Cardiovascular	1081	80.6
	Respiratory	252	18.8
	Others	9	0.7
ECMO mode	VA	1119	83.4
	VV	223	16.6
NYHA classification	n.a	79	5.9
	I	600	44.7
	II	314	23.4
	III	218	16.2
	IV	131	9.8
Post-operative	No	976	72.7
	Yes	366	27.3
IABP use	No	1104	82.3
	Yes	238	17.7
Extracorporeal CPR	No	819	61.0
	Yes	523	39.0
Infection before ECMO		950	70.8
		392	29.2
Pulmonary emboli		1336	99.6
		6	0.4
Dialysis before ECMO		1193	88.9
		149	11.1
LV ejection fraction	Missing	728	54.2
	< 55%	438	32.6
	≥ 55%	176	13.1
Central venous pressure	Missing	399	29.7
	≤ 12 mmHg	409	30.5
	> 12 mmHg	534	39.8
Bilirubin	Missing	307	22.9
	0–2	712	53.1
	2–5	226	16.8
	> 5	97	7.2
Blood urine nitrogen	Missing	208	15.5
	0–20	323	24.1
	20–50	550	41.0
	> 50	261	19.4
Lactate	Missing	274	20.4
	0–2	156	11.6
	2–10	532	39.6
	> 10	380	28.3
Prothrombin time	Missing	361	26.9
	0–2	873	65.1
	> 2	108	8.0
Creatine kinase	Missing	478	35.6
	0–200	347	25.9
	200–1000	318	23.7
	> 1000	199	14.8
Aspartate aminotransferase	Missing	413	30.8
	0–45	294	21.9
	45–135	286	21.3
	> 135	349	26.0
Creatine kinase MB form	Missing	507	37.8
	0–23	276	20.6
	23–69	279	20.8
	> 69	280	20.9
Troponin I	Missing	677	50.4
	0–23	564	42.0
	23–69	51	3.8
	69	50	3.7
Charlson Comorbidity Index	Missing	437	32.6
	0–14	544	40.5
	15–22	325	24.2
	22–47	36	2.7
APACHE Score	Missing	344	25.6
	0–14	304	22.7
	15–22	339	25.3
	22–47	355	26.5
SOFA Score	Missing	501	37.3
	1–9	596	44.4
	10–13	244	18.2
	14–24	1	0.1
LODS	Missing	522	38.9
	1–7	222	16.5
	8–11	341	25.4
	12–20	257	19.2
MODS	Missing	568	42.3
	0–7	260	19.4
	8–10	252	18.8
	11–19	262	19.5
SAPS3	Missing	522	38.9
	24–46	277	20.6
	47–58	287	21.4
	59–98	256	19.1
SAVE	Missing	913	68.0
	Class I	7	0.5
	Class II	52	3.9
	Class III	149	11.1
	Class IV	128	9.5
	Class V	93	6.9
**Outcome**	**Survival to discharge**	**506**	**37.7**
	**In-hospital death**	**836**	**62.3**

Charlson: Charlson Comorbidity Index, APACHE: Acute Physiologic Assessment and Chronic Health Evaluation II Scoring System, SOFA: Sequential Organ Failure Assessment Score, LODS: Logistic Organ Dysfunction System, MODS: Multiple Organ Dysfunction, SAPS3: Simplified Acute Physiology Score III, SAVE: The Survival After Veno-arterial ECMO Score. Outcome: the number and percentage of survival to discharge and in-hospital death

Under different partitions, RF and LR showed different discriminatory power (DP) in terms of area under the ROC curve. For partition 2, which used 934 cases for training, the training DP was 1.00 for RF and 0.80 for LR (Additional file 1: Table S3, upper panel, partition 2). The AUC of ROC became far more uniform when the resulting model was applied to the accompanying test set. For RF and LR, it was 0.70 and 0.74, respectively. Additional file 1: Table S3, upper panel, shows the same pattern regardless of the size of the training and test sets. Based on the parsimonious LR model, 11 clinical variables were significantly associated with in-hospital death. These 11 clinical variables include age, ventilation rate (VR), inotropic equivalent (IE), extracorporeal cardiopulmonary resuscitation (ECPR), infection before ECMO, systolic blood pressure (SBP), urine output (U/O), GCS, non-cardiovascular presentation, and bilirubin or creatine kinase levels. As shown in Table 3, a logit transformation of the probability of in-hospital death equals a linear combination of the 11 clinical variables. Both RF and LR models were constructed using parsimonious models with statistically significant variables. In both models, the in-hospital mortality rate showed a favorable DP. For RF, it was 0.73 and for LR, it was 0.76. (Additional file 1: Table S3, lower panel, partition 3).

**Table 3:** Logistic regression model used to predict in-hospital death with 11 clinical variables, including model description, odds ratio, *p* value, and 95% confidence interval

The model used to estimate the probability of in-hospital death				
Probability of in-hospital death = exp(*Y*)/(exp(*Y*) + 1), where				
*Y* = logit (probability of in-hospital death) =				
1.007				
+ 0.022 × Age				
+ 0.035 × Ventilation rate				
+ 0.007 × Inotropic equivalent				
+ 0.591 × Extracorporeal ECPR				
+ 0.767 × Infection before ECMO				
− 0.005 × Systolic blood pressure				
− 0.033 × Urine amount				
− 0.071 × Glasgow coma scale				
− 0.432 × [ECMO category = respiratory]				
− 1.721 × [ECMO category = others]				
− 0.573 × [Bil = missing]				
− 1.150 × [Bil = 0–2 mg/dL]				
− 0.733 × [Bil = 2–5 mg/dL]				
− 0.101 × [CKMB = missing]				
− 0.516 × [CKMB = 0–23 U/L]				
− 0.538 × [CKMB = 23–69 U/L]				

Variables	Odds ratio	*p*	95% confidence interval	
Age	1.022	< 0.001	1.014	1.03
Ventilation Rate	1.035	0.004	1.011	1.06
Inotropic equivalent	1.007	0.001	1.003	1.012
Extracorporeal CPR	1.806	< 0.001	1.356	2.407
Infection before ECMO	2.152	< 0.001	1.548	2.993
Systolic blood pressure	0.995	0.017	0.991	0.999
Urine amount	0.967	< 0.001	0.957	0.977
Glasgow coma scale	0.931	< 0.001	0.909	0.954
ECMO category		0.045		
Respiratory	0.649	0.045	0.425	0.991
Others	0.179	0.119	0.021	1.558
Cardiovascular	Reference			
Bilirubin		< 0.001		
Missing	0.564	0.077	0.299	1.063
0–2	0.317	< 0.001	0.174	0.577
2–5	0.48	0.025	0.253	0.913
> 5	Reference			
Creatine kinase MB		0.004		
Missing	0.904	0.599	0.622	1.316
0–23	0.597	0.01	0.403	0.884
23–69	0.584	0.007	0.396	0.862
> 69	Reference			

**Fig. 1:**
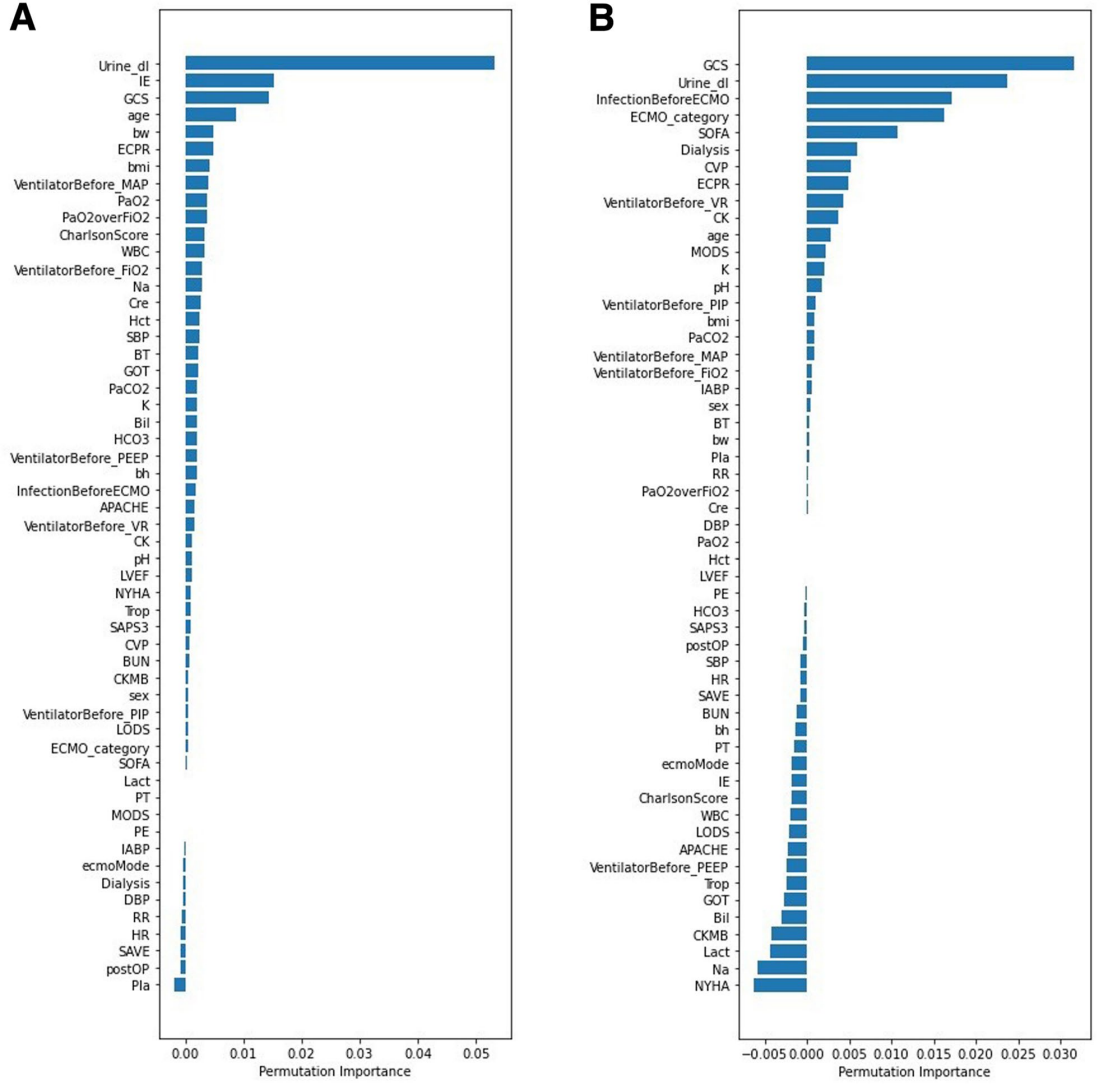
Ranking of the clinical features in the random forest (**A**) and logistic regression (**B**) models. bh: body height, bw: body weight, bmi: body mass index, NYHA: New York Hear Association functional class, ECMO: extracorporeal membrane oxygenation, Post OP: ECMO placed after an operation, IABP: Intra-aortic balloon pumping, ECPR: extracorporeal cardiopulmonary resuscitation, Infection: infection before ECMO, PE: pulmonary emboli, GCS: Glasgow coma scale, IE: inotropic equivalent, Dialysis: renal replacement therapy before ECMO, LVEF: left ventricular ejection fraction, VR: preset ventilation rate, FiO2: fraction of inspired oxygen, PIP: peak inspiratory pressure, PEEP: peak end-expiratory pressure, MAP: mean airway pressure, BT: body temperature, RR: respiratory rate, HR: heart rate, SBP: systolic blood pressure, DBP: diastolic blood pressure, CVP: central venous pressure, Urine_dl: 24-h urine amount (deciliter), pH: PH value, PaCO2: the partial pressure of carbon dioxide in the arterial blood, PaO2: the partial pressure of oxygen in the arterial blood, PaO2 over FiO2: PaO2/FiO2, HCO3: bicarbonate, Bil: total bilirubin, BUN: blood urine nitrogen, Cre: creatinine, Na: sodium, K: potassium, Lact: lactate, WBC: white blood cell, Hct: hematocrit, Pla: platelet, PT: prothrombin time, CK: creatine kinase, GOT: aspartate aminotransferase, CKMB: creatine kinase MB, Trop: troponin I, Charlson Score: Charlson Comorbidity Index, APACHE: Acute Physiologic Assessment and Chronic Health Evaluation II Scoring System, SOFA: Sequential Organ Failure Assessment Score, LODS: Logistic Organ Dysfunction System, MODS: Multiple Organ Dysfunction, SAVE: Survival after Veno-Arterial ECMO, SAPS3: Simplified Acute Physiology Score III

RF and LR models were used to examine the relative importance of all 55 features. The urine output (in deciliters) on the first day of ECMO institution was ranked as the first critical feature under the RF model (Fig. 1A), and again as the second important feature under the LR model (Fig. 1B). Although the ranking order of the other variables varied between the two models, the first 24 h urine output remained a powerful predictor of in-hospital mortality. In the subsequent survival analysis, this variable was selected as the major classifying feature [17].

**Fig. 2:**
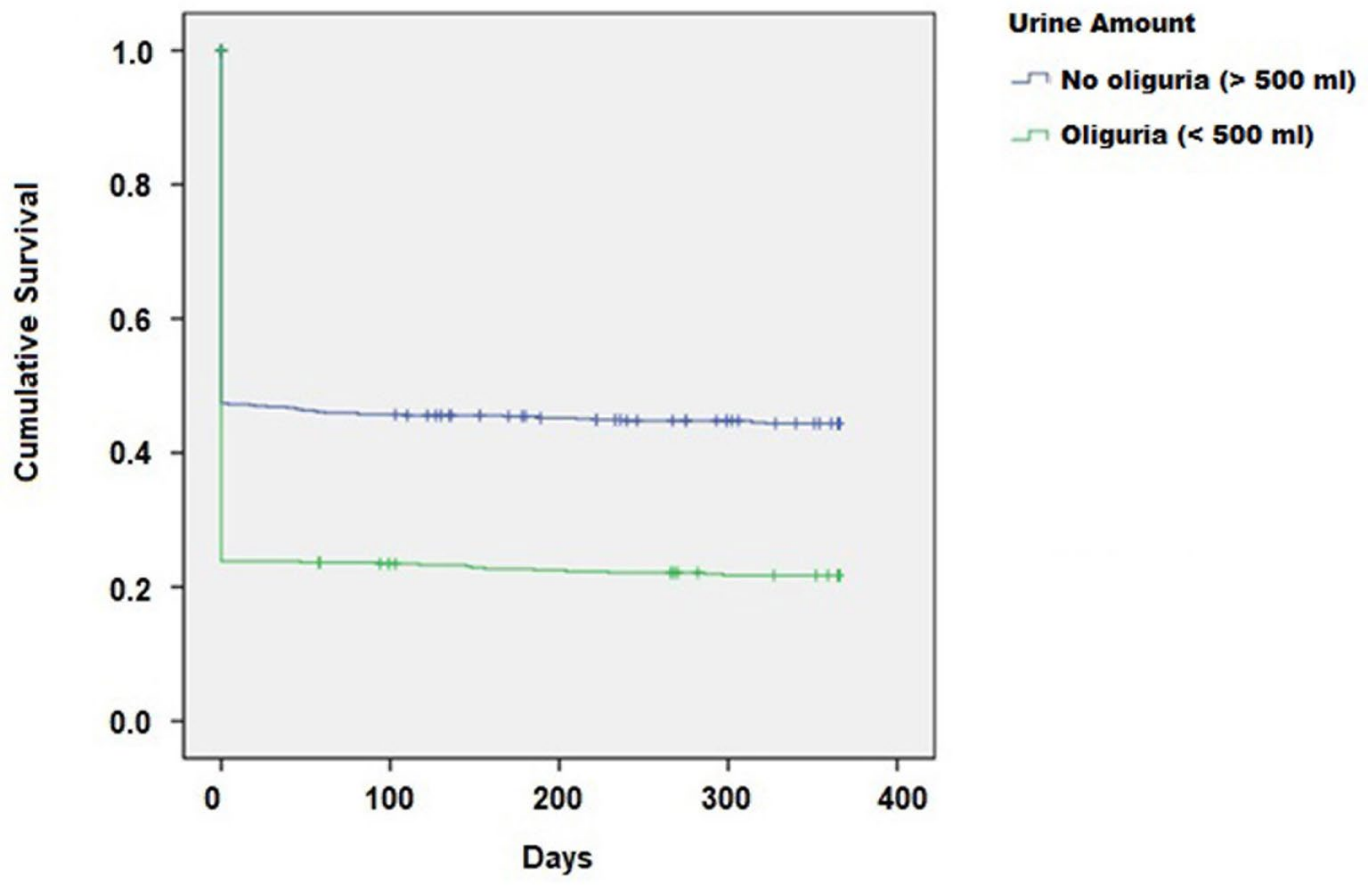
Kaplan–Meier curve comparing the patients with and without oliguria (urine output less than 500 ml)

There were three categories of urine output in the first 24 h: (1) normal (more than 10 dl), decreased (5 dl to 10 dl), and oliguric (less than 5 dl). A comparison of decreased urine group and normal urine group showed that the HR of in-hospital death was 1.005 (*p* = 0.970) for decreased urine group and 1.446 (*p* < 0.001) for oliguric group. Therefore, urine output less than 500 ml (i.e., 5 dl) seemed to affect in-hospital mortality. Our study compared patients with oliguria to those without oliguria by combining the first two categories (normal and decreased urine amount). As compared to patients whose initial urine output was greater than 5 dl, the HR for 1 year mortality in patients with oliguria was 1.445 (95% CI 1.265–1.650). Kaplan–Meier curve and log-rank test both revealed a significant difference between the overall mortality of the oliguric group and patients with normal and slightly reduced urine output (*p* < 0.001) (Fig. 2).

A multi-variable Cox regression model adjusted for other variables was also used to analyze 1 year mortality. As compared with the normal group, the HR for 1 year mortality was 1.016 (*p* = 0.90, 95% CI 0.798–1.000) for the decreased urine group and 1.295 (*p* < 0.001, 95% CI 1.116–1.502) for the oliguric group. When the patients were categorized only into oliguric and non-oliguric groups, the former (i.e., urine output < 5 dl) had a HR of 1.291 (*p* = 0.001, 95% CI 1.121–1.486) compared with the latter (urine output over 5 dl). For each 1 dl increase in urine output, the HR for 1 year mortality dropped by 0.986 (*p* < 0.001, 95% CI 0.979–0.992).

## Discussion

The final analysis of this study included 1342 patients with ECMO support. Compared to conventional approaches, this was by far the largest study of patients on ECMO evaluating prognostic indicators using machine learning algorithms of RF. One of the most influential features in predicting future death, among all anthropometric, hemodynamic, and laboratory variables, was urine output during the first 24 h after ECMO placement.

As part of the training process, RF uses many decision trees (DT) to determine a classifier. In both classification and regression tasks, the DT model is a non-parametric model based on a tree-like graph. A top-to-bottom tree-like structure represents the relationship between the recruiting features and the target variable. The DTs are very sensitive to the training data, so they are error-prone on the test data set. Different parts of the training data set are used for each DT to search for the most “votes,” which are combinations of features and thresholds which will result in the "most effective" separation between the two classes. Due to the fact that the RF algorithm interprets and gets the results from multiple DTs, it can reduce the variance that would result from considering just one DT alone [13]. In addition, we applied a series of solutions to reduce over-fitting in the RF algorithm, and found that the DP was comparable to that derived from LR model. On the other hand, parsimonious models based on select, clinically and statistically significant variables offer the prospect of numerical stability and generalizability, and as demonstrated here, better DP than non-parsimonious models. Thus, including a wider range of variables in the model regardless of their significance decreases the consistency between training and testing. The current analyses illustrate that concentrating only on key variables among all those available can produce a simpler model that also optimizes the DP.

According to both RF and LR models (Fig. 1A, B), urine output was one of the leading predictors, despite other features drifting their ranking scores irregularly. Furthermore, the amount of the 24 h urine output of the first day under ECMO institution determined the clinical outcomes of these critical patients. Oliguric patients were expected to have a poor prognosis. Clinical outcomes after ECMO may be determined by urine output due to global ischemia–reperfusion injury and renal hypoperfusion [18]. According to previous studies, oliguria is one of the earliest signs of insufficient organ perfusion [19]. Acute renal injury was reported in more than half of patients who were resuscitated following cardiopulmonary resuscitation (CPR) [18]. Oliguria could be used as a biomarker of acute kidney injury in critically ill patients [20]. A prior study also showed that in unselected critically ill patients, urine output obtained on ICU entry was associated with hospital mortality [21]. In patients receiving ECMO support, Combes et al. demonstrated that renal failure with a 24 h urine output less than 500 ml was significantly associated with ICU death (OR = 6.52) after ECMO implantation under cardiopulmonary resuscitation [22]. In out-of-hospital cardiac arrest patients receiving ECMO following CPR, Lee et al. found oliguria to be an independent risk factor for 30 day mortality [23]. According to Distelmaier et al. [19] 24 h urine output was a significant variable to predict 30 day and 2 year mortality after cardiovascular surgery. In comparison with previous studies (81, 23, and 205 in the studies by Combes, Lee, and Distelmaier, respectively), ours was by far the largest study and an entirely novel method for examining this issue [19, 22, 23].

In addition, urine output was routinely monitored in the ICU. Thus, urine output is a noninvasive, easily accessible, inexpensive, and ideal parameter for detecting high-risk mortality patients receiving ECMO support in time and intervening before adverse clinical outcomes occur. Low cardiac output (cardiogenic shock), systemic vasodilation (sepsis), and organ hypoperfusion may account for the decreased urine production under ECMO [19]. The condition may also be caused by decreased pump flow, impaired pulsatility, air embolization, hormonal disorders, or a change in platelet concentration [19, 24]. In addition, extracorporeal membranes and mechanical circuits may trigger inflammatory cascades with hypercoagulable states that could adversely affect microcirculation [19]. Due to the possibility that ECMO could trigger an acute inflammatory reaction, there might be capillary leakage and intravascular volume depletion resulting in acute tubular necrosis and oliguria [25].

## Limitations

It is possible that information might be lost if missing variables are replaced or if continuous variables are converted to categorical variables. To clarify this issue, we repeated the RF and LR analysis on 200 patients with complete data. The AUC for the in-hospital death remained near 0.75. The final DP did not appear to be altered by the pre-processing.

## Conclusions

In this study, 1342 patients undergoing ECMO support were enrolled, and independent predictors of in-hospital death were evaluated. The machine learning algorithm with RF and LR was used to find the most stable feature and robust DP in this prediction task. Our study found that oliguria, defined as urine output less than 500 ml within the first 24 h after ECMO implantation, was strongly associated with in-hospital death and 1 year mortality. To detect high-risk ECMO patients in advance and treat them promptly, urine output was a reliable and easy-to-use parameter.

### Supplementary Information

**Additional file 1: Table S1.** Variables and Definitions. **Table S2.** ECMO indications classified into three categories: cardiovascular, respiratory, and others. The numbers of VA and VV ECMO in each category were shown, and specific indications were reported. **Table S3.** Discriminatory power determined by the area under curve (AUC) in receiver operating characteristic curve (ROC) analyses using random forest and logistic regression model. Upper panel: non-parsimonious model when all variables were used. Lower panel: parsimonious model when only selected variables that were statistically significant were used. **Figure S1.** ROC curves for parsimonious models using random forest (Panel A) and logistic regression (Panel B) in the training set (70%, left column) and the test set (30%, right column).

## Data Availability

The data sets generated during and/or analyzed during the current study are not publicly available due to restrictions imposed by the institute and the government. This study's authors declare that all data supporting the conclusions are included in the paper and its supplementary information.

## Abbreviations

APACHE: Acute Physiologic Assessment and Chronic Health Evaluation
AST: Aspartate transaminase
AUC: Area under curve
CI: Confidence interval
CPR: Cardiopulmonary resuscitation
DP: Discriminatory power
ECMO: Extracorporeal membrane oxygenation
ELSO: Extracorporeal Life Support Organization
GCS: Glasgow Coma Scale
GOT: Aspartate transaminase
HR: Hazard ratio
IABP: Intra-aortic balloon pump
ICU: Intensive care unit
IE: Inotropic equivalent
LODS: Logistic Organ Dysfunction System
LR: Logistical regression
LVEF: Left ventricular ejection fraction
MODS: Multiple Organ Dysfunction
NTUH: National Taiwan University Hospital
NYHA: New York Heart Association
ROC: Receiver operating characteristic
SAPS III: Simplified acute physiology score III
SAVE: Survival After Veno-arterial ECMO
SOFA: Sequential Organ Failure Assessment
SVM: Support vector machine
VA: Veno-arterial
VV: Veno-venous
