# Supplementary material for: Urine output as one of the most important features in differentiating in-hospital death among patients receiving extracorporeal membrane oxygenation: a random forest approach
Source: Eur J Med Res. 2023 Sep 15;28:347. doi: 10.1186/s40001-023-01294-1 (PMC10503185; doi:10.1186/s40001-023-01294-1)
Supplement: Supplementary file 1 — Additional file 1: Table S1. Variables and Definitions. Table S2. ECMO indications classified into three categories: cardiovascular, respiratory, and others. The numbers of VA and VV ECMO in each category were shown, and specific indications were reported. Table S3. Discriminatory power determined by the area under curve (AUC) in receiver operating characteristic curve (ROC) analyses using random forest and logistic regression model. Upper panel: non-parsimonious model when all variables were used. Lower panel: parsimonious model when only selected variables that were statistically significant were used. Figure S1. ROC curves for parsimonious models using random forest (Panel A) and logistic regression (Panel B) in the training set (70%, left column) and the test set (30%, right column). [file 40001_2023_1294_MOESM1_ESM.docx]

**Supplementary Table 1.** Variables and Definitions

| Variable Name | Meaning | Data Types | Units | Definition of Categories |
| --- | --- | --- | --- | --- |
| bh | body height | continuous | cm |  |
| bw | body weight | continuous | kg |  |
| bmi | body mass index | continuous | kg/m^2^ |  |
| sex | sex | categorical | m or f |  |
| age | age | continuous | years |  |
| ECMO_category | ECMO category | categorical |  | cardiovascular, respiratory, or others |
| ecmoMode | ECMO mode | categorical |  | VA or VV |
| NYHA | NYHA functional class | categorical |  | null, I, II, III, IV |
| postOP | ECMO placed after an operation | categorical |  | yes or no |
| IABP | Intraaortic balloon pumping | categorical |  | yes or no |
| ECPR | ECMO assisted CPR | categorical |  | yes or no |
| Infection | infection before ECMO | categorical |  | yes or no |
| PE | pulmonary emboli | categorical |  | yes or no |
| GCS | Glasgow coma scale | continuous |  |  |
| IE | inotropic equivalent | continuous |  |  |
| Dialysis | renal replacement therapy before ECMO | categorical |  | yes or no |
| LVEF | left ventricular ejection fraction | categorical |  | missing, reduced (< 55), normal(>55) |
| VR | preset ventilation rate | continuous | per minute |  |
| FiO2 | FiO2 | continuous |  |  |
| PIP | peak inspiratory pressure | continuous | cmH_2_O |  |
| PEEP | peak end-expiratory pressure | continuous | cmH_2_O |  |
| MAP | mean airway pressure | continuous | cmH_2_O |  |
| BT | body temperature | continuous | degree Celsius |  |
| RR | respiratory rate | continuous | per minute |  |
| HR | heart rate | continuous | per minute |  |
| SBP | systolic blood pressure | continuous | mmHg |  |
| DBP | diastolic blood pressure | continuous | mmHg |  |
| CVP | central venous pressure | categorical | mmHg | missing, normal (CVP <=12), high (CVP > 12) |
| Urine_dl | 24-hour urine amount | continuous | dL |  |
| pH | pH | continuous |  |  |
| PaCO2 | PaCO2 | continuous | mmHg |  |
| PaO2 | PaO2 | continuous | mmHg |  |
| PaO2overFiO2 | PaO2/FiO2 | continuous | mmHg |  |
| HCO3 | bicarbonate | continuous | mEq/L |  |
| Bil | total bilirubin | categorical | mg/dL | missing, low(0-2), intermediate(2-5), high(>5) |
| BUN | blood urine nitrogen | categorical | mg/dL | missing, low(0-20), intermediate(20-50), high(>50) |
| Cre | creatinine | continuous | mg/dL |  |
| Na | sodium | continuous | mmole/L |  |
| K | potassium | continuous | mmole/L |  |
| Lact | lactate | categorical | mmole/L | missing, low(0-2), intermediate(2-10), high(>10) |
| WBC | white blood cell | continuous | per uL |  |
| Hct | hematocrit | continuous | % |  |
| Pla | platelet | continuous | 10^3^/uL |  |
| PT | prothrombin time | categorical | x | missing, normal(0-2), high(>2) |
| CK | creatine kinase | categorical | U/L | missing, low(0-200), intermediate(200-1000), high(>1000) |
| GOT | aspartate aminotransferase | categorical | U/L | missing, low(0-45), intermediate(45-135), high(>135) |
| CKMB | creatine kinase MB | categorical | U/L | missing, low(0-22.9), intermediate(23-68.9), high(> 69) |
| Trop | troponin I | categorical | ng/mL | missing, low(0-22.9), intermediate(23-68.9), high (>69) |
| CharlsonScore | Charlson Comorbidity Index | categorical |  | missing, low (0-4), intermediate (5-9), high (10-14) |
| APACHE | Acute Physiologic Assessment and Chronic Health Evaluation II Scoring System | categorical |  | missing, low(0-14), intermediate(15-22), high(22-47) |
| SOFA | Sequential Organ Failure Assessment Score | categorical |  | missing, low(1-9), intermediate(10-13), high(14-24) |
| LODS | Logistic Organ Dysfunction System | categorical |  | missing, low(1-7), intermediate(8-11), high(12-20) |
| MODS | Multiple Organ Dysfunction | categorical |  | missing, low(0-7), intermediate(8-10), high(11-19) |
| SAPS3 | Simplified Acute Physiology Score III | categorical |  | missing, low(24-46), intermediate(47-58), high(59-98) |
| inHospitalDeath | in-hospital death | TARGET VARIABLES |  | yes or no |

**Supplementary Table 2.** ECMO indications classified into three categories: cardiovascular, respiratory, and others. The numbers of VA and VV ECMO in each category were shown, and specific indications were reported.

| Categories | N | VA mode | VV mode | Specific Indications | n | % |
| --- | --- | --- | --- | --- | --- | --- |
| Cardiac Support | 1081 | 1063 | 18 | Acute myocarditis | 80 | 7.4 |
|  |  |  |  | Acute rejection | 21 | 1.9 |
|  |  |  |  | AMI | 290 | 26.8 |
|  |  |  |  | Other cardiac origins | 184 | 17 |
|  |  |  |  | Cardiomyopathy | 172 | 15.9 |
|  |  |  |  | Congenital heart disease | 3 | 0.3 |
|  |  |  |  | Endocrine heart disease | 7 | 0.6 |
|  |  |  |  | Pulmonary emboli | 33 | 3.1 |
|  |  |  |  | Right heart failure | 26 | 2.4 |
|  |  |  |  | Postcardiotomy | 160 | 14.8 |
|  |  |  |  | Septic shock | 105 | 9.7 |
| Respiratory Support | 252 | 53 | 199 | Airway stenosis | 2 | 0.8 |
|  |  |  |  | ARDS | 238 | 94.4 |
|  |  |  |  | Asthma | 2 | 0.8 |
|  |  |  |  | Respiratory others | 3 | 1.2 |
|  |  |  |  | Septic shock | 6 | 2.4 |
|  |  |  |  | Tracheal tumor/injury | 1 | 0.4 |
| Others | 9 | 3 | 6 | Others | 9 | 100 |

**Supplementary Table 3.** Discriminatory power determined by the area under curve (AUC) in receiver operating characteristic curve (ROC) analyses using random forest and logistic regression model. Upper panel: non-parsimonious model when all variables were used. Lower panel: parsimonious model when only selected variables that were statistically significant were used

|  | Partitions | Partition 1 | |  | Partition 2 | |  | Partition 3 | |  | Partition 4 | |
| --- | --- | --- | --- | --- | --- | --- | --- | --- | --- | --- | --- | --- |
|  |  | training | test |  | training | test |  | training | test |  | training | test |
|  | training to test ratio | 75% | 25% |  | 70% | 30% |  | 60% | 40% |  | 50% | 50% |
|  | n | 1002 | 340 |  | 934 | 408 |  | 793 | 549 |  | 656 | 686 |
| All variables | Random forest | 1.00 | 0.71 |  | 1.00 | 0.70 |  | 1.00 | 0.69 |  | 1.00 | 0.67 |
|  | Logistic regression | 0.80 | 0.73 |  | 0.80 | 0.74 |  | 0.81 | 0.72 |  | 0.83 | 0.70 |
| Selected Variables | Random forest | 1.00 | 0.69 |  | 1.00 | 0.67 |  | 1.00 | 0.73 |  | 1.00 | 0.70 |
|  | Logistic regression | 0.76 | 0.77 |  | 0.76 | 0.77 |  | 0.76 | 0.76 |  | 0.77 | 0.75 |

**A**


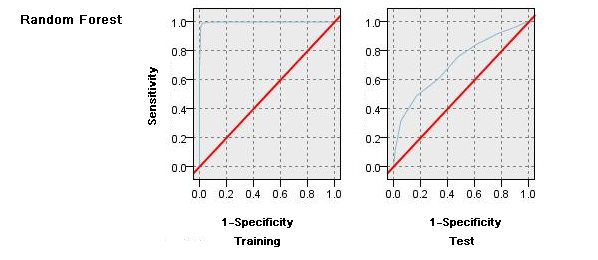


**B**


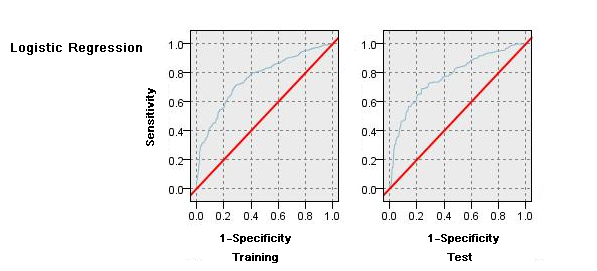


**Supplementary Figure 1.** ROC curves for parsimonious models using random forest (Panel A) and logistic regression (Panel B) in the training set (70%, left column) and the test set (30%, right column)
